# Oxidative Stress Biomarkers in Pediatric and Early-Stage Type 1 Diabetes: Toward Redox Phenotyping

**DOI:** 10.3390/antiox15081044

**Published:** 2026-08-21

**Authors:** Rahul Mittal, Alejandra Alberti, Roshini Shivakumar, Likhita Selvan, Vidhya Gupta, Ayushi Aggarwal, Venisha Patel, Carlos E. Blaschke, Farhad Alipour, Khemraj Hirani

**Affiliations:** 1 Diabetes Research Institute, University of Miami Miller School of Medicine, Miami, FL 33136, USA; alejandra.alberti001@mymdc.net (A.A.); shivakmr.roshini@gmail.com (R.S.); likhitha@unc.edu (L.S.); vidhya.gupta@pinecrest.edu (V.G.); ayushi-aggarwal@uiowa.edu (A.A.); venishagp2006@gmail.com (V.P.); cblaschke@med.miami.edu (C.E.B.); fna45@med.miami.edu (F.A.); 2 Division of Endocrinology, Diabetes, and Metabolism, Department of Medicine, University of Miami Miller School of Medicine, Miami, FL 33136, USA

**Keywords:** type 1 diabetes, pediatric diabetes, oxidative stress, redox phenotyping, antioxidant enzymes, lipid peroxidation, protein oxidation, DNA oxidation, inflammation, precision medicine

## Abstract

Type 1 diabetes (T1D) is characterized by autoimmune β-cell destruction, metabolic instability, and long-term susceptibility to vascular and renal complications. Oxidative stress has been widely implicated in these processes, but it is often described as a generalized consequence of hyperglycemia, not as a measurable and heterogeneous biological state. In this review, we propose exploratory redox phenotyping as a framework for characterizing oxidative stress in pediatric and early-stage T1D. This approach integrates biomarkers of antioxidant defense, lipid peroxidation, protein oxidation, DNA oxidation, and inflammation-associated redox activity to explore potentially distinct patterns of redox dysregulation. Key biomarker domains include enzymatic antioxidant systems, lipid-peroxidation products, protein- and DNA-oxidation markers, and inflammatory mediators, which may collectively characterize the intensity, molecular compartment, and possible clinical relevance of redox dysregulation. Across pediatric presymptomatic T1D, newly diagnosed disease, partial remission, and short-duration established T1D, longitudinal redox profiling may help distinguish stage-specific or transient metabolic responses from persistent oxidative dysregulation. These clinical phases are considered separately as they differ in immune activity, residual C-peptide secretion, glycemic exposure, and biological susceptibility. Redox phenotyping may also improve the design of antioxidant, mitochondrial, anti-inflammatory, and NRF2-targeted interventions by enabling biomarker-based patient selection. By defining oxidative stress as a stratifiable biological domain, this framework may advance precision risk assessment and guide more rational, targeted redox-directed therapies for children and individuals with early-stage T1D. At present, these proposed profiles remain hypothetical and require validation in prospective cohorts using standardized assays, reproducible thresholds, and clinically meaningful outcomes.

## 1. Introduction

Oxidative stress is defined as a disruption of redox homeostasis resulting from excess production of reactive oxygen species and reactive nitrogen species relative to endogenous antioxidant capacity [1,2,3,4,5]. In type 1 diabetes (T1D), this imbalance may occur during early disease and may reflect several interrelated mechanisms [6]. Chronic hyperglycemia increases mitochondrial substrate flux and enhances electron leakage from the respiratory chain, thereby increasing superoxide formation [7]. Glycemic variability may produce repeated acute changes in intracellular redox state [8]. Autoimmune inflammation exposes pancreatic β-cells, endothelial cells, and circulating immune cells to cytokine-mediated oxidative and nitrosative stress [9]. Altered lipid metabolism, endothelial activation, and impaired antioxidant buffering may further contribute to oxidative biomolecular modifications [6,10,11].

Oxidative stress has been extensively implicated in β-cell dysfunction, immune activation, endothelial injury, and the development of diabetic microvascular complications [10,12]. However, in most studies, oxidative stress has been treated as a generalized consequence of hyperglycemia rather than as a measurable and heterogeneous biological state [8,13]. This perspective has accompanied broad interest in antioxidant supplementation, although most studies have not incorporated biomarker-based patient selection [14,15]. Such an approach is biologically limited. The intensity, source, and molecular targets of oxidative stress are likely to differ according to age, disease duration, residual β-cell function, glycemic exposure, glycemic variability, adiposity, lipid phenotype, inflammatory status, and early tissue injury [8,16,17]. Therefore, oxidative stress in T1D should not be considered a uniform disease attribute.

For the purposes of this review, redox phenotyping is proposed as the systematic characterization of patients according to quantitative markers of oxidative damage, antioxidant defense, and inflammation-associated redox activity [6,18,19]. This framework moves beyond the nonspecific assessment of antioxidant status [20]. It is intended to explore whether patients can be distinguished according to demonstrable oxidative biomolecular injury, define the predominant redox compartment involved, and determine whether these patterns are associated with β-cell decline, metabolic instability, endothelial dysfunction, renal injury, retinal injury, or neuropathic risk [21,22,23,24]. In this context, oxidative stress could potentially be evaluated as a measurable component of disease heterogeneity rather than a broad mechanistic assumption.

In this review, populations represented across the early phases of T1D are considered clinically and biologically distinct groups, not a single homogeneous population. These groups include individuals with normoglycemic islet autoimmunity, individuals with autoantibody-positive dysglycemia, patients at or shortly after clinical diagnosis, individuals undergoing partial clinical remission, and patients with established T1D of relatively short duration. These disease phases differ in autoimmune activity, β-cell functional reserve, endogenous C-peptide secretion, magnitude and duration of hyperglycemic exposure, exogenous insulin dependence, metabolic stability, and susceptibility to acute or cumulative tissue injury. Each of these factors may independently influence oxidative stress, antioxidant capacity, and the interpretation of redox biomarkers. Accordingly, evidence from presymptomatic, newly diagnosed, remission-phase, and short-duration T1D populations is discussed separately whenever the relevant clinical characteristics are adequately reported. The term “early-stage T1D” is used only as a broad descriptor encompassing the early phases of disease and does not imply clinical or biological equivalence among these populations.

**Figure 1:**
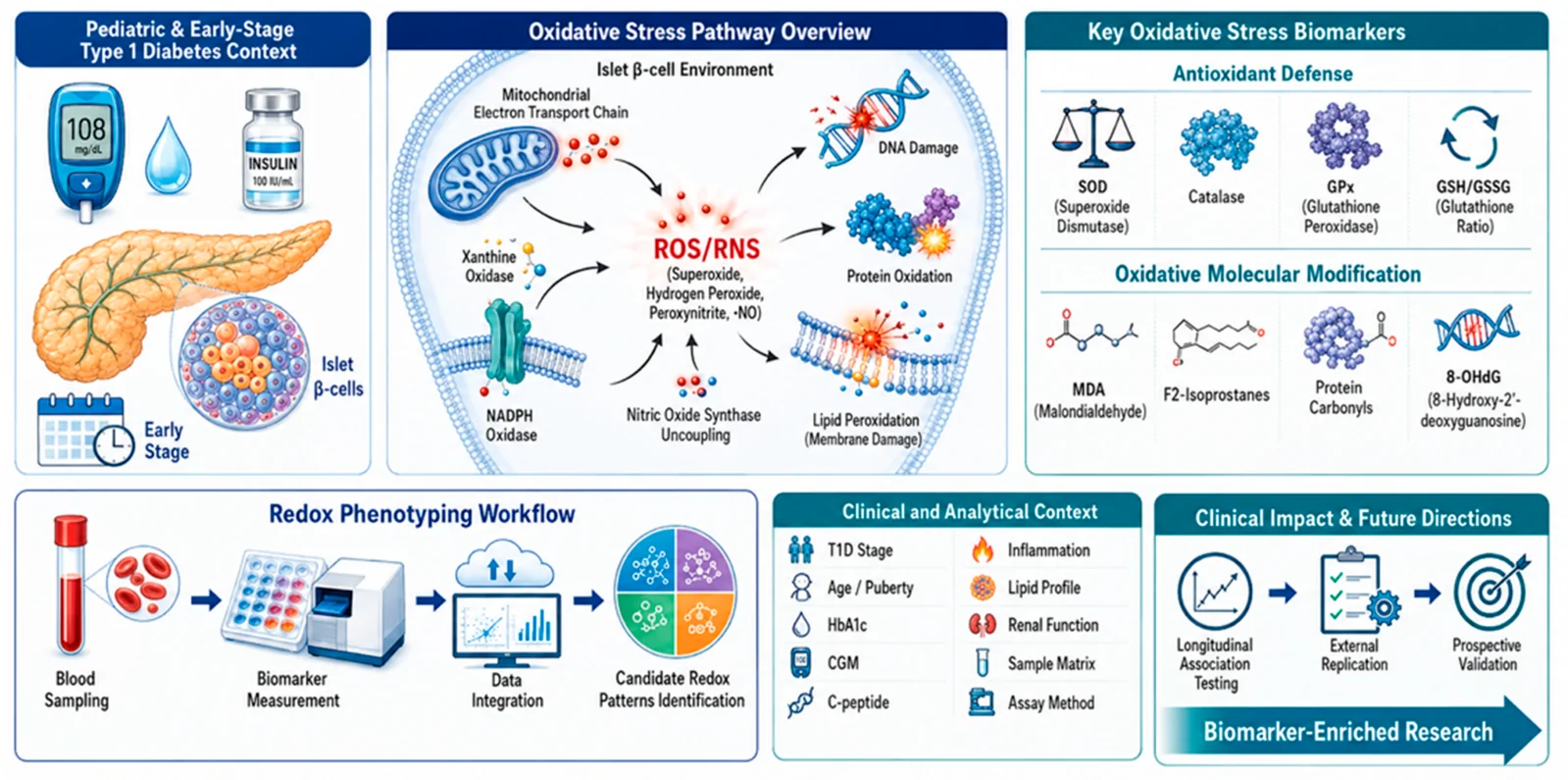
Conceptual framework for redox profiling in pediatric and early-stage type 1 diabetes. Mitochondrial dysfunction, NADPH oxidase activity, inflammatory signaling, and glycemic variability may contribute to increased generation of reactive oxygen and nitrogen species in β-cells and other metabolically or immunologically active compartments, resulting in oxidative modification of DNA, proteins, and lipids. Representative biomarkers include 8-hydroxy-2′-deoxyguanosine, malondialdehyde, F2-isoprostanes, protein carbonyls, the GSH/GSSG ratio, superoxide dismutase, and catalase. Integrated assessment of these markers together with disease stage and established clinical variables may facilitate the identification of candidate redox patterns and assessment of associations with disease progression and clinically relevant outcomes. The proposed patterns are exploratory and hypothesis-generating, requiring analytical standardization, independent replication, and prospective validation before use in prognostic stratification, treatment selection, or biomarker-guided intervention. Created in BioRender. Abbreviations: 8-OHdG, 8-hydroxy-2′-deoxyguanosine; GSH, reduced glutathione; GSSG, oxidized glutathione; MDA, malondialdehyde; NADPH, nicotinamide adenine dinucleotide phosphate; ROS, reactive oxygen species; SOD, superoxide dismutase; T1D, type 1 diabetes.

The objective of this review is to provide an integrated perspective on oxidative stress as a measurable and biologically stratifiable component of disease heterogeneity in T1D (Figure 1). It brings together findings on enzymatic antioxidant defenses, lipid, protein, and DNA oxidation, inflammation–redox interactions, pediatric-specific studies, and longitudinal clinical outcomes within a unified conceptual framework. This perspective enables oxidative stress biomarkers to be viewed as complementary biological signatures reflecting distinct patterns of redox dysregulation and their relationship with β-cell dysfunction, glycemic instability, inflammatory activity, and early vascular injury. The framework is intended to generate testable hypotheses concerning biologically distinct patterns of redox dysregulation and their possible associations with disease progression and clinical outcomes. Its utility for patient classification, longitudinal monitoring, risk prediction, or therapeutic stratification remains to be established in prospective, independently validated cohorts.

## 2. Literature Search

This article was developed as a structured narrative review integrating clinical and mechanistic evidence on oxidative stress biomarkers in pediatric and early-stage T1D. A comprehensive literature search was conducted in PubMed (MEDLINE), ScienceDirect, Scopus, and Embase. The literature search covered publications from 1 January 2000 to 31 March 2026. Articles available online ahead of print or in press at the time of the search were considered eligible and were cited using their subsequently assigned publication details where applicable. Google Scholar was used as a supplementary source to identify additional relevant publications and citation-linked articles. Reference lists of eligible primary studies, systematic reviews, consensus statements, and clinical guidelines were also examined to identify publications not retrieved through the database searches.

The search strategy combined controlled vocabulary, including Medical Subject Headings and Emtree terms, with free-text keywords related to T1D and redox biology. Principal search concepts included “type 1 diabetes,” “pediatric,” “child,” “adolescent,” “islet autoimmunity,” “stage 1 type 1 diabetes,” “stage 2 type 1 diabetes,” “new-onset type 1 diabetes,” “partial remission,” “oxidative stress,” “redox homeostasis,” “antioxidant defense,” “superoxide dismutase,” “catalase,” “glutathione peroxidase,” “glutathione,” “lipid peroxidation,” “malondialdehyde,” “F2-isoprostanes,” “oxidized low-density lipoprotein,” “protein oxidation,” “protein carbonyls,” “advanced oxidation protein products,” “nitrotyrosine,” “DNA oxidation,” “8-hydroxy-2′-deoxyguanosine,” “inflammation,” “mitochondrial dysfunction,” and “NRF2.”

Publications were considered eligible when they examined oxidative stress pathways, antioxidant-defense systems, redox biomarkers, inflammation-associated redox activity, mitochondrial dysfunction, or redox-directed interventions relevant to T1D. Evidence from human observational studies, prospective cohorts, clinical trials, systematic reviews, meta-analyses, consensus documents, and experimental investigations was considered. Priority was assigned to studies involving children and adolescents and to populations with presymptomatic stage 1 or stage 2 T1D, newly diagnosed stage 3 T1D, partial clinical remission, or short-duration established T1D. Adult T1D studies were included when they provided clinically relevant evidence concerning biomarker–outcome associations, residual C-peptide secretion, vascular or renal complications, analytical performance, or longitudinal outcomes that were insufficiently examined in pediatric cohorts. Animal studies, isolated human-islet investigations, cellular models, and other in vitro experiments were included to support mechanistic interpretation and therapeutic plausibility.

Studies focused exclusively on type 2 diabetes or unrelated disorders were not considered direct evidence for pediatric T1D. Selected studies from other populations were retained only when they addressed fundamental redox mechanisms, biomarker chemistry, preanalytical determinants, assay validation, or analytical limitations directly relevant to the interpretation of oxidative stress biomarkers. Conference abstracts, non-peer-reviewed reports, and publications lacking sufficient methodological or population-level information were used cautiously and were not treated as definitive clinical evidence.

Evidence was classified according to population and experimental context before synthesis. Pediatric human evidence included studies conducted predominantly in participants younger than 18 years or studies reporting pediatric findings separately. Adult human evidence included clinical investigations conducted predominantly in participants aged 18 years or older. Mixed-age studies were classified according to the population contributing the relevant findings; studies without separately reported pediatric results were not considered direct pediatric evidence. Experimental evidence included animal models, isolated human islets, primary-cell studies, cell lines, and other in vitro systems. Pediatric human studies were considered the most directly applicable source of clinical evidence, adult human studies were interpreted as supportive evidence requiring cautious extrapolation, and experimental studies were used to establish biological plausibility without being considered evidence of clinical validity.

The evidence was synthesized qualitatively due to substantial heterogeneity in participant age, T1D stage, disease duration, metabolic stability, residual β-cell function, biological matrix, sample handling, assay platform, biomarker units, comparator groups, and outcome definitions. Greater interpretive weight was assigned to prospective and longitudinal studies, clearly characterized populations, standardized analytical procedures, appropriate adjustment for confounding variables, replication across independent cohorts, and associations with clinically meaningful outcomes. Small cross-sectional studies, incompletely defined disease populations, and investigations using insufficiently standardized assays were interpreted more cautiously. No quantitative meta-analysis or pooled effect estimation was undertaken.

## 3. Biological Rationale: Mechanisms Linking T1D to Distinct Redox Phenotypes

The biological rationale for redox phenotyping in T1D derives from the convergence of metabolic, inflammatory, mitochondrial, and endothelial stress pathways [9,25]. These pathways do not operate with equal intensity in all patients [6,26]. They also do not produce identical molecular damage [21,27]. Hyperglycemia can increase mitochondrial substrate pressure and enhance electron leakage from the respiratory chain [28]. This promotes superoxide generation and the secondary formation of hydrogen peroxide, hydroxyl radicals, and reactive nitrogen species [25,29]. At the same time, autoimmune inflammation activates cytokine-dependent signaling in β-cells, immune cells, and endothelial cells [30,31]. These processes may alter antioxidant enzyme activity, glutathione availability, nitric oxide signaling, and mitochondrial integrity [9,32]. The resulting redox state is therefore not a single biochemical event [8]. It is an integrated phenotype shaped by glycemic exposure, immune activity, cellular antioxidant capacity, and tissue susceptibility [10,21].

Pancreatic β-cells are especially relevant to this framework because they are metabolically active and relatively vulnerable to oxidative stress [9,17]. β-cells also have comparatively limited antioxidant defense capacity, particularly with respect to catalase and glutathione peroxidase activity, which may reduce their ability to neutralize hydrogen peroxide and lipid hydroperoxides under sustained stress [33,34]. This vulnerability provides a mechanistic basis for linking oxidative stress to impaired insulin gene expression, defective glucose-stimulated insulin secretion, loss of β-cell identity, and cell death [9,17]. It also supports the idea that redox biomarkers may reflect ongoing β-cell stress during early T1D, especially when residual C-peptide is still present [17,35].

Hyperglycemia contributes to redox imbalance through several interdependent biochemical pathways [7,13]. Increased intracellular glucose flux can overload mitochondrial oxidative phosphorylation and increase electron donation to the respiratory chain [28,29]. Excess superoxide can then interact with nitric oxide to form peroxynitrite or be converted to hydrogen peroxide through superoxide dismutase [10,25]. Hyperglycemia also promotes advanced glycation end-product formation, protein kinase C activation, polyol pathway flux, and hexosamine pathway activity [36,37]. These pathways can impair endothelial function, modify proteins, alter gene expression, and increase inflammatory signaling [9,24]. In clinical T1D, these mechanisms are likely intensified by glycemic variability [8,26]. Repeated transitions between normoglycemia and hyperglycemia may generate recurrent oxidative shifts rather than a stable redox state [8,38]. This may explain why two patients with similar HbA1c values can differ in oxidative biomarker profiles.

**Figure 2:**
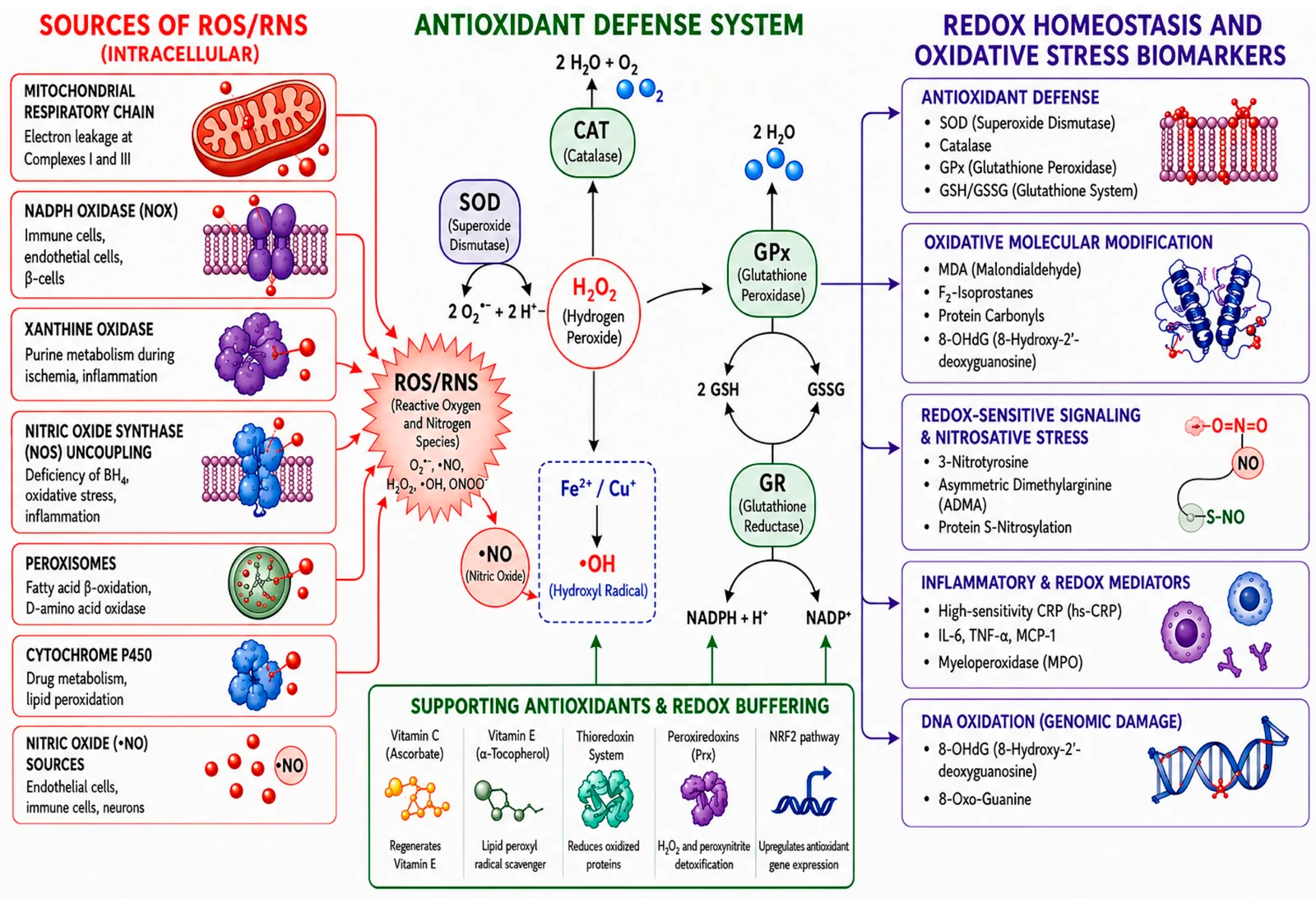
Intracellular sources of reactive oxygen species, antioxidant defense pathways, and biomarkers of redox homeostasis and oxidative stress relevant to type 1 diabetes. Mitochondrial electron leakage, NADPH oxidases, xanthine oxidase, peroxisomal metabolism, uncoupled endothelial nitric oxide synthase, and cytochrome P450 contribute to the generation of superoxide and related reactive species. Superoxide dismutase converts superoxide to hydrogen peroxide, which is detoxified by catalase and the glutathione peroxidase–glutathione reductase system, with additional contributions from thioredoxin, peroxiredoxins, vitamins C and E, and NRF2-regulated antioxidant responses. Incomplete detoxification may contribute to lipid peroxidation, protein oxidation, DNA oxidation, nitrosative stress, and inflammation-associated redox activity. Representative biomarkers reflecting antioxidant defense, redox status, oxidative molecular injury, and inflammation-associated redox activity are shown. The proposed relationships between biomarker patterns, biological pathways, and clinical characteristics are conceptual and hypothesis-generating. They do not represent validated redox phenotypes and require analytical standardization, independent replication, and prospective validation. Created in BioRender.

Mitochondrial electron leakage is not the only intracellular source of reactive species relevant to T1D. NADPH oxidase isoforms in β-cells, leukocytes, and vascular cells can generate superoxide in response to hyperglycemia, cytokines, and receptor-mediated activation. Xanthine oxidase may contribute to superoxide and hydrogen-peroxide generation during purine metabolism and endothelial stress, whereas the oxidation of tetrahydrobiopterin or limited L-arginine availability can uncouple nitric oxide synthase, shifting the enzyme from nitric oxide production toward superoxide generation. These sources may generate overlapping biomarker patterns. NADPH-oxidase activation and nitric-oxide-synthase uncoupling may be associated with altered superoxide handling, reduced nitric oxide bioavailability, nitrotyrosine formation, and endothelial activation, whereas xanthine-oxidase activity may contribute to urate-associated and peroxide-related redox signals. As commonly measured circulating biomarkers cannot uniquely identify the responsible enzymatic source, source attribution should remain provisional unless supported by pathway-specific assays or intervention studies. The major intracellular sources of ROS, antioxidant defense pathways, and representative oxidative damage biomarkers are summarized in Figure 2.

Autoimmune inflammation provides a second major source of redox stress [30]. During islet inflammation, β-cells are exposed to cytokines such as interleukin-1β, interferon-γ, and tumor necrosis factor-α [39,40]. These mediators can induce nitric oxide production, mitochondrial dysfunction, endoplasmic reticulum stress, and apoptotic signaling [41]. Immune cells also generate reactive species as part of inflammatory activation [42]. In this setting, oxidative stress is not merely a consequence of hyperglycemia [30,39]. It may participate in immune-mediated β-cell injury before or around the time of clinical diagnosis [43,44]. This distinction is important for early-stage disease [45]. A child with active islet autoimmunity and modest dysglycemia may show a different redox profile from a child with established insulin deficiency and prolonged metabolic exposure [6,27]. Redox phenotyping may therefore help separate inflammation-dominant oxidative stress from hyperglycemia-dominant oxidative stress [21,27].

Mitochondrial dysfunction can amplify redox injury as oxidative modification of respiratory-chain proteins, mitochondrial DNA, and membrane lipids may reduce electron-transport efficiency and further increase reactive-species generation [9]. Circulating biomarkers can indicate systemic mitochondrial-associated stress but cannot establish islet, immune-cell, endothelial, or renal tissue origin [32,35].

The antioxidant response also varies across individuals and disease phases [26]. Superoxide dismutase, catalase, glutathione peroxidase, glutathione reductase, thioredoxin, peroxiredoxins, and reduced glutathione participate in coordinated antioxidant defense [32,46]. Increased activity of an antioxidant enzyme may represent adaptive induction in response to oxidant exposure [47]. It should not automatically be interpreted as adequate protection. Conversely, reduced enzyme activity may reflect depletion, glycation-related dysfunction, micronutrient insufficiency, impaired transcriptional regulation, or loss of cellular reserve [48]. Glutathione-dependent systems are particularly relevant because they link peroxide detoxification to cellular redox buffering [32]. Altered glutathione status may indicate impaired capacity to neutralize hydrogen peroxide and lipid hydroperoxides [46]. Therefore, antioxidant biomarkers require contextual interpretation. Their significance depends on whether oxidative damage markers are also elevated, whether inflammation is present, and whether metabolic stress is ongoing [26,27].

NRF2 is a major transcriptional regulator of endogenous antioxidant and cytoprotective responses [49]. Under basal conditions, NRF2 is restrained by KEAP1-dependent degradation. Under oxidative or electrophilic stress, NRF2 can accumulate and induce genes involved in glutathione synthesis, detoxification, peroxide reduction, xenobiotic metabolism, and cellular repair [49,50]. The experimental and review literature supports a role for NRF2 in β-cell survival and stress adaptation [49]. However, NRF2 biology is complex. Acute or regulated activation may be protective in models of β-cell stress, whereas chronic, excessive, or context-inappropriate activation may have different effects depending on tissue and disease state [51]. This complexity reinforces the need for redox phenotyping before considering NRF2-directed strategies. Biomarker evidence of impaired antioxidant response, oxidative damage, or inflammation-linked redox activation would provide a stronger rationale for targeted modulation than diagnosis of T1D alone [49,50].

Lipid metabolism provides another route to heterogeneous oxidative phenotypes [52]. Even in T1D, dyslipidemia, altered lipoprotein composition, and glycation of apolipoproteins can increase susceptibility to lipid oxidation [53,54]. Oxidized lipids and lipid peroxidation products can modify membranes, impair endothelial function, and amplify inflammatory signaling [52]. Malondialdehyde, F2-isoprostanes, and oxidized low-density lipoprotein therefore reflect more than nonspecific oxidative injury [55]. They may identify patients in whom redox stress is concentrated in membranes, lipoproteins, and vascular compartments [52,56]. Such a pattern could be particularly relevant for investigation in adolescents and in patients with higher adiposity, poorer glycemic control, or early cardiometabolic risk factors [53,57,58]. It may differ mechanistically from a phenotype dominated by β-cell inflammatory stress or glutathione depletion.

Protein and DNA oxidation represent more durable forms of molecular injury [52,59]. Protein carbonylation, advanced oxidation protein products, and nitrotyrosine indicate oxidative or nitrative modification of enzymes, albumin, structural proteins, and signaling molecules [48,60]. These changes may alter protein function and contribute to vascular or renal pathology [21,55]. Oxidative DNA lesions, including 8-hydroxy-2′-deoxyguanosine, indicate nucleobase oxidation and may reflect sustained oxidative exposure, inflammatory activation, or impaired repair [59,61]. These biomarkers are not specific to T1D and require careful interpretation. They may be influenced by infection, renal handling, exercise, diet, assay platform, and sample processing. Their value is therefore strongest when they are interpreted with glycemic metrics, inflammatory markers, antioxidant indices, and clinical context [21,26].

These mechanisms support multimarker assessment but do not establish clinically valid redox classes [62]. The major oxidative stress biomarker domains and their potential biological and clinical interpretation within a redox phenotyping framework are summarized in Table 1.

## 4. Enzymatic Antioxidants: SOD, Catalase, Glutathione Peroxidase, and Redox Defense Capacity

SOD, catalase, GPx, and glutathione reductase constitute an integrated enzymatic antioxidant network that regulates superoxide and peroxide metabolism. SOD catalyzes the dismutation of superoxide anion to hydrogen peroxide and molecular oxygen. The resulting hydrogen peroxide is subsequently detoxified by catalase, GPx, peroxiredoxins, and related peroxide-reducing systems, whereas glutathione reductase uses NADPH to regenerate reduced glutathione from oxidized glutathione and thereby sustain glutathione-dependent peroxide reduction [68,80,81]. These measurements reflect antioxidant-defense capacity and adaptive redox responses rather than oxidative molecular injury itself. Increased enzyme activity may indicate compensatory induction in response to increased oxidant generation, whereas reduced activity may result from oxidative or glycation-related inactivation, the depletion of glutathione or other required substrates, micronutrient insufficiency, impaired transcriptional regulation, or loss of functional reserve. Accordingly, SOD, catalase, GPx, and glutathione reductase should not be interpreted as isolated high or low values. Their biological significance depends on concurrent evidence of lipid, protein, or DNA oxidation and on the clinical and analytical context, including glycemic exposure and variability, inflammatory activity, renal function, nutritional and micronutrient status, biological matrix, preanalytical handling, and assay methodology.

### 4.1. Superoxide Dismutase and Superoxide Handling

Superoxide dismutase is a central enzyme in the early response to superoxide production. Its major isoforms include cytosolic copper–zinc superoxide dismutase, mitochondrial manganese superoxide dismutase, and extracellular superoxide dismutase. These isoforms occupy different cellular and extracellular compartments. This compartmentalization is relevant in T1D as oxidative stress can arise in mitochondria, vascular endothelium, immune cells, erythrocytes, and extracellular spaces. A circulating or erythrocyte SOD measurement therefore reflects systemic antioxidant activity rather than direct islet-specific redox status.

Superoxide is produced during mitochondrial electron transport and by oxidant-generating enzymes such as NADPH oxidases [82]. In hyperglycemic conditions, increased substrate availability can increase electron pressure within the respiratory chain. This favors superoxide formation, especially when mitochondrial coupling is impaired. In endothelial cells, excess superoxide reduces nitric oxide bioavailability and contributes to impaired vasodilation. In immune cells, superoxide participates in inflammatory responses [83]. In β-cells, superoxide and derived reactive species can interfere with mitochondrial ATP production and glucose-stimulated insulin secretion. These mechanisms support the use of SOD activity as an indicator of superoxide handling in T1D [84].

SOD activity alone is insufficient to define oxidative risk because the enzyme converts superoxide into hydrogen peroxide. If downstream peroxide removal by catalase, glutathione peroxidase, peroxiredoxins, and related systems is inadequate, increased SOD activity may coexist with persistent oxidative injury. SOD should therefore be interpreted together with peroxide-detoxifying enzymes, glutathione status, and molecular-damage markers [27].

### 4.2. Catalase and Hydrogen Peroxide Clearance

Catalase is a heme-containing enzyme that rapidly converts hydrogen peroxide into water and oxygen. It is highly efficient at high hydrogen peroxide concentrations and is enriched in peroxisomes. Its function is especially relevant when hydrogen peroxide production exceeds the capacity of glutathione- and thioredoxin-dependent systems. In diabetes, hydrogen peroxide is generated from superoxide dismutation, mitochondrial oxidant leakage, peroxisomal metabolism, inflammatory cell activity, and oxidase-dependent reactions. Catalase therefore represents a key downstream component of peroxide clearance [85].

Catalase activity in T1D may reflect either adaptive peroxide clearance or impaired antioxidant capacity, depending on the clinical and biochemical context. Its interpretation should therefore account for disease duration, glycemic variability, inflammatory status, and concurrent markers of oxidative injury [86,87].

Catalase is also relevant to β-cell biology because pancreatic β-cells have relatively limited peroxide-detoxifying capacity. This contributes to their sensitivity to oxidative and cytokine-mediated stress. Circulating catalase does not directly measure β-cell catalase activity, but it provides systemic information about peroxide handling [88,89].

Recent pediatric data support continued interest in catalase as a redox biomarker. A 2025 pediatric case–control study comparing young children with T1D, transient hyperglycemia, and healthy controls measured oxidative stress and antioxidant-related parameters. The study reported altered oxidative stress responses in pediatric T1D, including differences in malondialdehyde and glutathione peroxidase-related relationships [27]. Another study reported higher catalase activity in children with T1D and proposed catalase as a candidate marker requiring validation. These findings are consistent with catalase as a component of the pediatric T1D redox response, but they do not establish catalase as a diagnostic or prognostic marker on its own [90].

Within a redox-phenotyping model, catalase is best interpreted as an index of hydrogen-peroxide clearance. Elevated catalase with low molecular-damage markers may indicate effective adaptation, whereas elevated catalase with persistent lipid or protein oxidation suggests incomplete compensation. Low catalase combined with high SOD suggests impaired downstream peroxide handling; concomitantly low catalase and GPx with elevated injury markers suggests broader antioxidant-defense failure [80].

### 4.3. Glutathione Peroxidase, Glutathione Cycling, and Selenium Dependence

Glutathione peroxidase is a family of selenium-dependent and non-selenium-dependent enzymes that reduce hydrogen peroxide and lipid hydroperoxides. GPx activity depends on reduced glutathione as an electron donor. Oxidized glutathione is then converted back to reduced glutathione by glutathione reductase using NADPH. This pathway links peroxide detoxification to cellular metabolism, micronutrient status, and redox buffering capacity. It is especially important for detoxifying lipid hydroperoxides, which can propagate membrane injury and generate reactive aldehydes such as malondialdehyde.

In T1D, GPx activity should be interpreted together with reduced and oxidized glutathione, glutathione reductase activity, NADPH availability, selenium status, glycemic exposure, inflammation, and molecular-damage markers. This combined assessment can distinguish preserved antioxidant reserve from substrate depletion, impaired glutathione recycling, or compensatory enzyme activation [26,27].

Pediatric evidence supports the relevance of glutathione-dependent defense in early T1D. A study reported increased malondialdehyde and altered relationships among GPx, glutathione, selenium, zinc, and lipid variables in children with T1D compared with transient hyperglycemia. These findings support the interpretation of GPx within a broader metabolic and micronutrient context [27].

As selenium availability influences selenium-dependent GPx activity, pediatric studies should assess GPx together with glutathione status and relevant micronutrient indices whenever feasible.

### 4.4. Erythrocyte, Plasma, Serum, and Tissue Compartments

The biological interpretation of antioxidant enzymes depends strongly on the sample compartment. Erythrocyte SOD, catalase, and GPx primarily reflect red-cell antioxidant capacity and systemic exposure. Plasma or serum measurements may be influenced by extracellular enzyme release, inflammation, hemolysis, and sample handling, whereas leukocyte assays provide information about immune-cell redox biology. None of these circulating compartments directly measure pancreatic islet oxidative stress. Therefore, findings should be described as circulating or systemic unless tissue-specific evidence is available.

Preanalytical and analytical standardization is essential. Hemolysis, processing delay, storage temperature, freeze–thaw cycles, anticoagulant choice, fasting status, recent exercise, acute infection, pubertal stage, and metabolic instability can alter results. Studies should also distinguish enzyme activity from protein abundance, transcript expression, and gene expression because these measurements represent different biological properties and should not be used interchangeably.

## 5. Lipid Peroxidation Markers: Malondialdehyde, F2-Isoprostanes, Oxidized LDL, and Membrane–Lipoprotein Redox Injury

Lipid-peroxidation biomarkers reflect oxidative modification of membranes and lipoproteins rather than antioxidant-defense capacity. In T1D, lipid oxidation may arise from hyperglycemia, glycemic variability, altered lipoprotein composition, inflammation, and endothelial dysfunction. MDA, F2-isoprostanes, oxidized LDL, lipid hydroperoxides, and 4-hydroxynonenal provide partly different information, but none of them are tissue-specific or sufficient to define a clinical phenotype independently.

### 5.1. Malondialdehyde as a Lipid Peroxidation Product

Malondialdehyde is one of the most widely measured lipid peroxidation products in diabetes research. It is generated during the oxidative degradation of polyunsaturated fatty acids and is also formed during prostaglandin and thromboxane biosynthesis. It is present in human tissues, serum, and platelets, and it has long been used as an accessible indicator of lipid peroxidation. Its popularity reflects practical advantages. Assays are relatively inexpensive, and blood samples are easy to obtain. Pediatric studies can incorporate MDA measurement without the technical demands of mass spectrometry.

The biological interpretation of MDA requires precision. MDA is not simply a passive marker. It is a reactive aldehyde. It can form adducts with proteins and DNA. These adducts can alter protein structure, enzyme activity, immune recognition, and genomic stability. This feature links lipid peroxidation to protein oxidation and DNA damage. It also explains why MDA elevation can indicate a broader oxidative injury state rather than isolated membrane oxidation. However, MDA is not specific to one tissue or pathway. It can reflect oxidative modification of membranes, lipoproteins, platelets, and inflammatory compartments [91].

MDA is especially useful in pediatric redox phenotyping when interpreted with antioxidants and metabolic markers. Increased MDA with preserved catalase, glutathione peroxidase, and glutathione suggests lipid oxidative burden with retained defense capacity. Increased MDA with low glutathione or reduced GPx activity indicates impaired detoxification of lipid hydroperoxides. Increased MDA with elevated CRP, IL-6, or TNF-α suggests inflammation-linked lipid peroxidation. Increased MDA together with dyslipidemia or oxidized LDL may be compatible with a lipoprotein-associated oxidative pattern [92]. These combinations are more informative than MDA alone.

Assay limitations are substantial. Many clinical studies measure thiobarbituric acid reactive substances rather than MDA-specific products. The thiobarbituric acid assay can be affected by non-MDA aldehydes, sugars, amino acids, bilirubin, and sample handling. MDA is also influenced by diet, lipid composition, storage conditions, hemolysis, and processing delay. For this reason, high-performance liquid chromatography- or mass spectrometry-based approaches provide better specificity than colorimetric thiobarbituric acid assays. Studies that use MDA should describe the analytical platform, sample type, fasting status, storage conditions, and quality-control procedures [93]. Without this information, between-study comparisons are limited.

In pediatric T1D, MDA is best used as an accessible component of a lipid-peroxidation panel rather than as a diagnostic marker. Pairing it with GPx, glutathione, micronutrient status, lipid profile, HbA1c, and CGM-derived variability improves mechanistic interpretation.

### 5.2. F2-Isoprostanes and In Vivo Lipid Oxidation

F2-isoprostanes are prostaglandin-like compounds formed by non-enzymatic free radical-mediated peroxidation of arachidonic acid. They are chemically stable and measurable in plasma, urine, and other biological fluids. A substantial body of evidence supports F2-isoprostanes as reliable indices of lipid peroxidation in vivo, and several reviews describe them as among the most accurate available biomarkers of oxidative lipid injury. This gives them an important position in redox phenotyping. They provide a more specific measure of lipid peroxidation than many older colorimetric assays. The strength of F2-isoprostanes lies in their mechanism of formation and analytical specificity. They are generated independently of cyclooxygenase pathways through free radical attack on arachidonic acid [94]. Their stability allows measurement in stored samples when appropriate protocols are followed. Urinary measurement offers a non-invasive approach, which is attractive in pediatric research. Plasma measurement can reflect circulating lipid peroxidation more directly but is more sensitive to sample processing. Mass spectrometry-based quantification remains the most rigorous approach. Immunoassays are easier to use but can differ in specificity and comparability.

For T1D, F2-isoprostanes are conceptually valuable because they quantify systemic oxidative lipid injury without relying on nonspecific aldehyde chemistry. They can be used to evaluate whether oxidative stress is present in early disease, whether it tracks with glycemic variability, and whether it predicts vascular or renal risk. Their use in children is feasible, especially through urine collection. However, interpretation still requires clinical context. F2-isoprostanes can be affected by age, diet, adiposity, smoking exposure, infection, exercise, renal function, and medication exposure. Pediatric studies should therefore collect these covariates.

For intervention studies, elevated baseline F2-isoprostanes may identify a lipid-peroxidation phenotype and provide a pathway-specific endpoint. This biomarker-enriched design is preferable to enrolling unselected T1D cohorts and expecting uniform antioxidant effects.

### 5.3. Oxidized LDL and Lipoprotein-Centered Oxidative Injury

Oxidized low-density lipoprotein represents oxidative modification of circulating LDL particles. It links oxidative stress to atherogenic biology, endothelial dysfunction, macrophage activation, and vascular inflammation. LDL particles are vulnerable to oxidative modification when exposed to reactive oxygen species, reactive nitrogen species, glycation, and inflammatory enzymes [95]. In diabetes, hyperglycemia and glycation alter lipoprotein structure and increase susceptibility to oxidation. Dyslipidemia, insulin resistance, adiposity, and chronic inflammation can further intensify lipoprotein oxidative modification.

In T1D, oxidized LDL is important because cardiovascular risk begins to evolve before clinical cardiovascular disease becomes apparent. Atherosclerotic processes and vascular risk may begin to develop during childhood in individuals with T1D. Children and adolescents with T1D can develop early endothelial dysfunction, altered arterial stiffness, and unfavorable lipid changes [67]. Oxidized LDL provides a mechanistic link between redox stress and early vascular risk. It differs from MDA and F2-isoprostanes because it reflects oxidation of a specific circulating lipoprotein fraction rather than global lipid peroxidation [96].

The interpretation of oxidized LDL requires integration with the lipid profile. High oxidized LDL in the setting of elevated LDL cholesterol, non-HDL cholesterol, triglycerides, or low HDL cholesterol suggests a lipoprotein-centered redox phenotype [97]. High oxidized LDL, despite having a normal LDL-cholesterol concentration, may provide information about oxidative lipoprotein modification that is not captured by LDL concentration alone. This distinction is important for T1D. Standard lipid panels quantify lipid burden. Oxidized LDL reflects oxidative transformation of that burden. The two measures are related but not interchangeable [92].

Oxidized LDL also interacts with inflammation. It can activate endothelial cells, stimulate monocyte adhesion, promote macrophage uptake through scavenger receptors, and contribute to foam cell formation. These events are central to early atherogenesis. In pediatric redox phenotyping, oxidized LDL therefore belongs in an inflammation–vascular risk panel. Within the proposed framework, oxidized LDL could be interpreted alongside CRP, cytokines, endothelial markers, lipid profile, glycemic metrics, blood pressure, BMI, and albuminuria. Isolated oxidized LDL elevation is harder to interpret without these clinical and biochemical variables.

Analytical issues remain important. Oxidized LDL assays differ in antibody specificity and target epitopes. Some assays detect oxidized phospholipid epitopes. Others detect modified apolipoprotein B or broader oxidized LDL particles. Results across platforms are not always comparable. Preanalytical handling, lipid-lowering therapy, renal function, acute inflammation, and sample storage can affect value. Therefore, oxidized LDL is best used in carefully designed research protocols or as part of mechanistic panels rather than as a standalone clinical test.

### 5.4. Lipid Hydroperoxides and 4-Hydroxynonenal

Lipid hydroperoxides are primary products of lipid peroxidation. They form early in the oxidative chain reaction and can decompose into secondary products such as MDA and 4-hydroxynonenal. Their measurement provides insight into active lipid oxidation before downstream aldehydes accumulate. However, lipid hydroperoxides are chemically unstable and sensitive to sample handling. This limits their use in large pediatric cohorts unless protocols are rigorous.

4-hydroxynonenal is a reactive α, β-unsaturated aldehyde formed mainly from peroxidation of ω-6 polyunsaturated fatty acids. It is more than a damage marker. It can form covalent adducts with proteins, alter enzyme activity, modify signaling pathways, and influence inflammation. At low concentrations, 4-hydroxynonenal participates in adaptive signaling. At higher concentrations, it contributes to cytotoxicity. This dose-dependent biology complicates interpretation but increases mechanistic relevance.

In T1D, 4-hydroxynonenal is relevant to mitochondrial function, β-cell stress, endothelial dysfunction, and immune signaling. β-cells are sensitive to lipid aldehydes because mitochondrial metabolism and membrane integrity are essential for glucose-stimulated insulin secretion. Endothelial cells exposed to lipid aldehydes can develop impaired nitric oxide signaling and increased inflammatory adhesion molecule expression. These mechanisms support inclusion of lipid aldehydes in advanced redox panels. However, pediatric clinical evidence remains less developed for 4-hydroxynonenal than for MDA.

Lipid hydroperoxides and 4-HNE are currently best regarded as research-grade biomarkers. They may add mechanistic detail to panels containing MDA, F2-isoprostanes, oxidized LDL, GPx, glutathione, and inflammatory markers, but analytical complexity and limited pediatric reference data restrict first-line use.

## 6. Protein Oxidation: Structural Macromolecular Modification and Functional Redox Injury

Protein oxidation represents an important form of macromolecular redox injury in T1D. Compared with short-lived reactive species, some oxidatively modified proteins are sufficiently stable to provide an integrated measure of recent or cumulative oxidative exposure. This makes them useful for identifying sustained redox disturbance. Proteins regulate catalysis, transport, immune recognition, cytoskeletal integrity, receptor signaling, extracellular matrix structure, and vascular function [69]. Oxidative modification can therefore produce direct biological consequences. In T1D, protein oxidation arises through ROS-mediated amino acid oxidation, RNS-mediated nitration, lipid aldehyde adduction, and glycoxidative reactions [44]. These mechanisms generate a heterogeneous spectrum of modified proteins rather than a single molecular lesion.

Several protein compartments are susceptible to oxidative modification in T1D. Albumin, hemoglobin, apolipoproteins, complement proteins, immunoglobulins, endothelial proteins, renal matrix proteins, and intracellular enzymes can undergo oxidation, nitration, carbonylation, or cross-linking [77]. These modifications can impair ligand binding, reduce enzymatic activity, alter protein conformation, increase aggregation, and change immunogenicity.

### 6.1. Protein Carbonyls and Carbonyl Stress

Protein carbonyls are established markers of oxidative protein modification [68]. Carbonyl groups form through direct oxidation of lysine, arginine, proline, and threonine residues. They also arise through reactions with reactive carbonyl species generated during lipid peroxidation and glycoxidation. This dual origin is highly relevant in T1D, where oxidative stress, lipid aldehyde formation, and glucose-derived carbonyl stress coexist. Carbonylated proteins are relatively stable. Their accumulation reflects an imbalance between macromolecular injury and proteolytic clearance through proteasomal degradation, autophagy, extracellular turnover, and renal handling [60]. Carbonylation can impair the function and clearance of circulating, intracellular, and structural proteins. These effects link oxidative protein modification to endothelial dysfunction, immune activation, and early renal stress. In redox phenotyping, protein carbonyls therefore identify accumulated macromolecular injury rather than compensatory antioxidant activation [88].

Protein carbonyls should be interpreted with lipid peroxidation, glycemic variability, inflammation, and renal indices. Elevation with MDA or F2-isoprostanes indicates propagation from lipid oxidation to protein modification. Elevation with AOPPs or nitrotyrosine indicates broader oxidative and nitrative protein injury [71]. Elevation with albuminuria or endothelial markers suggests tissue-level relevance. Acute infection, DKA, and marked metabolic instability should therefore be documented as potential confounders when interpreting protein-carbonyl measurements.

### 6.2. AOPPs and Inflammatory Oxidant Chemistry

AOPPs are oxidized and cross-linked protein derivatives generated during exposure of plasma proteins to strong oxidants. Albumin is a major substrate. Myeloperoxidase-derived chlorinated oxidants contribute substantially to AOPP formation. This biochemical origin gives AOPPs a specific interpretation. They reflect protein oxidation in the context of inflammatory oxidant activity rather than nonspecific oxidative protein damage [80].

AOPPs are relevant to T1D because they link inflammation, protein oxidation, and vascular or renal stress. Experimental studies have associated AOPPs with activation of oxidative pathways, inflammatory mediator expression, endothelial dysfunction, and renal cellular injury. Their interpretation is strongest when integrated with CRP, IL-6, TNF-α, albuminuria, endothelial markers, and renal function. Elevated AOPPs with inflammatory activation indicate immune-associated protein oxidation. Elevated AOPPs with albuminuria or vascular dysfunction indicate a redox profile with potential tissue-level relevance.

AOPPs also help distinguish oxidative protein injury from glycation-dominant modification. AGEs and AOPPs can coexist in diabetes, but they are not equivalent. AGEs reflect nonenzymatic glycation and glycoxidation. AOPPs reflect oxidant-mediated protein cross-linking, especially in inflammatory oxidative environments. Concurrent assessment can separate glycation-predominant injury from inflammatory protein oxidation [70]. This distinction is important for redox phenotyping because the upstream mechanisms and potential intervention targets differ.

### 6.3. Nitrotyrosine and Nitrative Protein Modification

Nitrotyrosine reflects tyrosine nitration and indicates RNS-associated protein modification. Its formation is closely linked to nitric oxide–superoxide interactions and peroxynitrite-related chemistry. This is relevant in T1D because endothelial nitric oxide signaling, mitochondrial superoxide production, immune-cell activation, and inflammatory nitric oxide generation can occur simultaneously [98]. Nitrotyrosine therefore captures nitrative stress rather than nonspecific oxidative damage.

Nitrative modification can alter protein structure, enzymatic activity, receptor signaling, and phosphorylation-dependent pathways [72]. In endothelial compartments, nitrative stress can reduce nitric oxide bioavailability and impair vasodilatory signaling. In immune compartments, it reflects inflammatory redox activation. In β-cell stress models, cytokine-induced nitric oxide production and mitochondrial oxidant generation contribute to nitrative injury [96]. These mechanisms support inclusion of nitrotyrosine in advanced redox panels, especially when vascular or inflammation-linked phenotypes are being evaluated.

Nitrotyrosine is most informative when interpreted with inflammatory and vascular markers. Elevated nitrotyrosine together with IL-6, TNF-α, or CRP may support an inflammation-associated nitrative pattern. Elevation with oxidized LDL or endothelial dysfunction markers supports vascular redox imbalance. Within the proposed framework, concurrent elevation of nitrotyrosine with inflammatory, vascular, or protein-oxidation markers could be evaluated as a candidate inflammation-associated nitrative pattern. This pattern provides greater mechanistic specificity than a broad classification of protein oxidation.

## 7. DNA Oxidation: Genomic Redox Injury, Repair Response, and Cellular Stress Signaling

DNA oxidation reflects redox-mediated modification of genetic material. It represents a potentially consequential injury domain because DNA integrity is required for transcriptional fidelity, cellular survival, mitochondrial function, and tissue repair. In T1D, DNA oxidation is biologically plausible because hyperglycemia, mitochondrial dysfunction, cytokine exposure, lipid aldehydes, and inflammatory oxidant production can affect both nuclear and mitochondrial genomes [73]. DNA oxidation does not measure antioxidant capacity. It indicates that reactive intermediates have reached genomic substrates and generated lesions requiring repair. Oxidative DNA lesions can alter base pairing, activate DNA repair pathways, induce cell-cycle arrest, stimulate stress-response signaling, and promote apoptosis when repair capacity is insufficient. These processes are relevant in β-cells, endothelial cells, renal cells, and immune cells. β-cells require intact mitochondrial and nuclear signaling for insulin synthesis and secretion. Endothelial cells require genomic stability for vascular homeostasis. Renal cells require repair capacity during sustained metabolic stress. Immune cells undergo metabolic activation and redox remodeling during autoimmune inflammation [99]. DNA oxidation therefore provides a mechanistic link between systemic redox stress and cellular resilience.

### 7.1. 8-OHdG and 8-oxodG as Guanine Oxidation Markers

Guanine is highly susceptible to oxidative modification because of its low redox potential. Oxidation generates 8-OHdG and 8-oxodG, which are widely used markers of oxidative DNA injury [100]. These biomarkers reflect formation and repair of oxidized guanine lesions. They can be measured in urine, plasma, serum, leukocyte DNA, or tissue. Each matrix provides distinct biological information.

Urinary 8-OHdG is useful in pediatric studies because collection is non-invasive. It reflects systemic oxidative DNA turnover and repair product excretion. Plasma or serum measurements reflect circulating oxidized nucleosides or DNA-related products. Leukocyte DNA measurements provide information about immune-cell genomic redox stress. Tissue measurement provides compartment-specific information but is rarely feasible in pediatric T1D. These matrices should not be considered interchangeable.

8-OHdG and 8-oxodG are not T1D-specific. Their concentration can be influenced by infection, exercise, diet, environmental exposures, inflammation, renal handling, and assay platform. Their scientific value lies in contextual interpretation. Persistent elevation during stable clinical conditions indicates sustained genomic oxidative burden. Interpretation should therefore consider matrix, renal function, inflammatory state, clinical stability, and assay method.

### 7.2. DNA Repair and Urinary Biomarker Interpretation

Oxidative DNA lesions are continuously removed by repair systems. BER is the principal pathway for correction of oxidized bases. OGG1 recognizes oxidized guanine lesions and initiates repair. Repair products can be excreted in urine. Urinary 8-OHdG therefore reflects oxidative DNA turnover rather than lesion formation alone. This distinction is essential for interpretation. Increased urinary excretion can indicate higher oxidative lesion burden, increased repair activity, or both.

This biology affects redox phenotyping. Elevated urinary 8-OHdG with preserved antioxidant defenses and low macromolecular injury markers suggests active repair under moderate oxidative pressure. Elevated urinary 8-OHdG with lipid peroxidation, protein oxidation, reduced GSH, or inflammatory activation may be consistent with a broader multimolecular oxidative-injury pattern. Low urinary 8-OHdG does not exclude DNA injury if repair capacity or renal excretion is altered [74]. Interpretation therefore requires renal indices, inflammatory markers, antioxidant status, and clinical stability.

Creatinine normalization is commonly used for urinary biomarkers. In children, this approach is influenced by age, sex, muscle mass, pubertal stage, hydration, and renal function. Longitudinal within-patient trends are therefore more informative than isolated cross-sectional values. Standardized collection timing, hydration context, renal assessment, and assay methodology are necessary for rigorous pediatric studies.

### 7.3. Nuclear and Mitochondrial DNA Compartments

Oxidative injury to nuclear DNA and mtDNA has different biological implications [101]. Nuclear DNA damage can alter transcription, activate checkpoint pathways, and contribute to apoptosis or senescence. mtDNA damage can impair oxidative phosphorylation, reduce ATP generation, and increase mitochondrial ROS production. This distinction is highly relevant in T1D because mitochondrial function regulates β-cell glucose sensing, endothelial nitric oxide homeostasis, and immune-cell activation [102]. mtDNA is vulnerable because it is located close to the respiratory chain and has distinct packaging and repair characteristics. Damage to mtDNA-encoded respiratory chain components can reduce electron transport efficiency and amplify ROS generation. In β-cells, this can impair stimulus-secretion coupling. In endothelial cells, it can impair vascular homeostasis. In immune cells, it can modify inflammatory metabolism. Circulating 8-OHdG does not establish whether nuclear DNA or mtDNA is the dominant source. Compartment-specific assays are required for that distinction.

For pediatric T1D, systemic DNA oxidation markers remain practical, but tissue attribution should be avoided unless directly measured. Future pediatric T1D studies should evaluate 8-OHdG or 8-oxodG alongside mitochondrial-function indices, C-peptide, CGM metrics, inflammatory mediators, renal indices, and vascular markers. This allows systemic genomic oxidative burden to be placed within a biologically coherent framework.

## 8. Inflammation–Redox Panels: Immune-Metabolic Integration and Phenotypic Stratification

Inflammation and redox biology are mechanistically interdependent in T1D [103]. Autoimmune activity, cytokine signaling, metabolic stress, endothelial activation, and leukocyte responses shape the oxidative environment. ROS and RNS influence inflammatory transcription, antigen modification, vascular signaling, and cell death pathways. Isolated oxidative stress markers therefore provide incomplete biological information when inflammatory context is absent [10]. Integrated inflammation–redox panels address this by measuring immune activation and molecular oxidative injury within the same framework.

These panels improve mechanistic resolution. Lipid peroxidation without inflammatory activation differs from lipid peroxidation with cytokine elevation and protein nitration [94]. Increased antioxidant enzyme activity without oxidative molecular injury differs from increased enzyme activity with inflammatory protein oxidation. DNA oxidation with systemic inflammation differs from DNA oxidation during acute metabolic instability [10]. These distinctions are essential for defining reproducible redox phenotypes rather than nonspecific oxidative stress states [104].

The inflammatory layer commonly includes hsCRP, IL-6, TNF-α, IL-1β, IFN-γ, selected chemokines, adhesion molecules, and leukocyte activation markers [105]. The redox layer can include SOD, CAT, GPx, GSH, MDA, F2-isoprostanes, oxidized LDL, protein carbonyls, AOPPs, nitrotyrosine, oxidized albumin, 8-OHdG, and 8-oxodG [106]. The clinical layer should include HbA1c, CGM metrics, C-peptide, lipid profile, BMI, BP, UACR, eGFR, and disease duration [67]. This architecture links immune activity, oxidative injury, antioxidant reserve, and clinical phenotype [107].

### 8.1. Cytokine-Associated Redox Signaling

Cytokines provide a direct mechanistic connection between autoimmunity and redox injury. In T1D, β-cells and immune cells are exposed to IL-1β, IFN-γ, and TNF-α. Their effects intersect with redox biology because cytokine signaling increases ROS and RNS generation while reducing cellular tolerance to oxidative burden [108]. Systemic inflammatory mediators also affect peripheral metabolic and vascular compartments [105]. TNF-α can impair insulin signaling, promote endothelial activation, and alter mitochondrial function. IL-6 links innate immune activity with hepatic acute-phase signaling and metabolic regulation [109]. hsCRP reflects downstream systemic inflammatory tone [105]. These markers are not disease-specific, but their integration with redox injury markers provides mechanistic information. Cytokine elevation with nitrotyrosine supports RNS-associated injury. Cytokine elevation with AOPPs supports inflammatory protein oxidation. Cytokine elevation with oxidized LDL supports vascular-lipoprotein redox activation [10].

In pediatric T1D, cytokine-associated redox patterns require clinical context [67]. Intercurrent infection, puberty, adiposity, glycemic instability, and autoimmune activity can influence inflammatory markers [110]. A cytokine abnormality without oxidative molecular injury has different significance from cytokine activation accompanied by lipid, protein, or DNA oxidation [104]. Prospective studies should determine whether persistent co-elevation during stable clinical conditions defines a reproducible inflammation–redox pattern.

### 8.2. Endothelial Inflammation–Redox Coupling

The endothelium is a major site of inflammation–redox interaction in T1D [103]. Hyperglycemia, oxidized lipids, cytokines, and glycated proteins impair nitric oxide signaling and increase oxidant generation [12]. Superoxide reduces nitric oxide bioavailability and contributes to peroxynitrite formation. Peroxynitrite drives nitrative protein modification [111]. Cytokines increase adhesion molecule expression and promote leukocyte-endothelial interaction. These processes connect metabolic exposure, inflammatory activation, and vascular oxidative injury [109].

A vascular inflammation–redox profile can include oxidized LDL, nitrotyrosine, AOPPs, hsCRP, IL-6, TNF-α, endothelial adhesion molecules, BP, lipid profile, and UACR [67]. Each component captures a different dimension of vascular stress. Oxidized LDL reflects lipoprotein modification. Nitrotyrosine reflects nitric oxide–superoxide imbalance. AOPPs reflect inflammatory protein oxidation. hsCRP and cytokines reflect systemic inflammatory tone. UACR and BP provide clinical context for vascular and renal involvement [103]. Such an integrated profile may provide greater biological context than an isolated oxidative-stress measurement, although its incremental clinical value remains to be established.

This approach is relevant in pediatric disease because vascular injury begins as a subclinical process. Children with T1D usually lack overt cardiovascular disease, yet endothelial dysfunction and renal stress can develop early [112]. Inflammation–redox panels can identify biochemical patterns preceding clinical events [67]. Their purpose is biological stratification, not premature diagnosis of complications.

### 8.3. Renal Inflammation–Redox Biology

The kidney is highly exposed to metabolic, hemodynamic, inflammatory, and oxidative stress in T1D. Glomerular endothelial cells, podocytes, mesangial cells, and tubular epithelial cells encounter hyperglycemia, filtered proteins, cytokines, oxidized lipids, AGEs, and oxidized plasma proteins [65]. Redox injury can alter filtration barrier integrity, tubular signaling, extracellular matrix turnover, and inflammatory recruitment. Renal indices are therefore essential anchors for inflammation–redox panels.

Protein oxidation and DNA oxidation are particularly relevant to renal redox biology. AOPPs indicate oxidized plasma protein burden and inflammatory oxidant exposure. Oxidized albumin reflects altered extracellular antioxidant function and modified filtered protein load. 8-OHdG and 8-oxodG indicate systemic or renal-associated genomic oxidative turnover, depending on matrix and renal context [113]. These markers require interpretation with UACR, eGFR, BP, and inflammatory mediators [114]. This combined approach could be evaluated as a candidate renal redox pattern with greater biological coherence than an isolated urinary or plasma marker [115].

In pediatric T1D, renal inflammation–redox assessment is valuable because conventional nephropathy markers become abnormal after earlier biological changes have occurred. Persistent co-elevation of inflammatory markers, oxidized proteins, DNA oxidation products, and renal indices suggests a stronger renal redox signal than any isolated abnormality [107]. Longitudinal evaluation can determine whether such profiles predict progression, remain stable, or normalize with improved metabolic control.

## 9. Pediatric T1D: Developmental Redox Biology and Early Disease Stratification

### 9.1. Developmental Context of Redox Regulation in Pediatric T1D

Pediatric T1D develops within a changing biological system. Growth, puberty, immune maturation, body composition, diet, physical activity, sleep, and intercurrent infections all influence metabolic regulation and redox homeostasis [116]. These variables distinguish pediatric T1D from adult disease and require a developmental interpretation of oxidative biomarkers [67]. A redox profile in a preschool child near diagnosis cannot be interpreted in the same biological frame as a profile in a pubertal adolescent with longer disease duration, insulin resistance, and evolving cardiometabolic risk. Age and developmental stage therefore function as biological modifiers of redox phenotype rather than background demographic variables [114].

Early childhood is characterized by rapid growth, variable dietary intake, frequent infections, and changing immune responsiveness [114]. These factors affect inflammatory tone, antioxidant substrate availability, and metabolic stability. In young children with T1D, glycemic excursions are often pronounced because insulin requirements are small, food intake is unpredictable, and counterregulatory responses differ from those in older patients [117]. These glycemic fluctuations can influence redox signaling even when average glycemic indices appear acceptable. In this setting, redox biomarkers may provide complementary information about biological stress that is not represented by mean glycemic exposure alone.

Puberty introduces additional complexity. Growth hormone, sex steroids, changing adiposity, and pubertal insulin resistance alter glucose handling and inflammatory biology. Adolescents often experience higher insulin requirements, wider glycemic variability, and less favorable lipid profiles. These changes can modify oxidative burden and antioxidant reserve. A pubertal increase in redox biomarkers should therefore be interpreted with Tanner stage, insulin dose, body composition, lipid profile, and CGM metrics [118]. Without developmental context, redox phenotypes can be misclassified.

Pediatric redox phenotyping should also account for nutritional and micronutrient biology. GPx activity depends on selenium availability. SOD activity is influenced by copper, zinc, and manganese-dependent isoforms. GSH metabolism depends on amino acid availability, NADPH generation, and cellular metabolic state. Dietary quality, gastrointestinal comorbidity, selective eating, food insecurity, and supplement exposure can influence these variables [118]. In children, antioxidant capacity is therefore shaped by both disease biology and developmental nutrition. This makes paired assessments of redox biomarkers and nutritional context scientifically necessary.

### 9.2. Early-Stage Disease and the Partial Remission Interval

Presymptomatic stage 1 and stage 2 T1D, newly diagnosed stage 3 T1D, and the subsequent partial-remission period represent clinically related yet biologically distinct phases for redox phenotyping [119]. These populations should be evaluated separately in analytical models, as they differ in glycemic status, exposure to exogenous insulin, residual β-cell function, intensity of immune activity, and potential influence of acute metabolic decompensation on circulating redox biomarkers. Before and near diagnosis, immune activity, β-cell stress, metabolic instability, and residual insulin secretion coexist. This period differs from long-standing insulin-dependent disease. The redox profile during this interval can reflect active immune-metabolic transition rather than established complication biology [120]. Measurements obtained at diagnosis, after stabilization, during partial remission, and after remission decline can define temporal redox trajectories.

The partial remission interval is especially important. During this phase, residual β-cell function contributes to improved glycemic stability and reduced exogenous insulin requirements. C-peptide remains detectable in many patients. Immune activity and β-cell stress continue, but metabolic burden is reduced relative to acute presentation. Redox biomarkers during partial remission can therefore help distinguish reversible metabolic stress from persistent immune-redox activation. If oxidative injury markers normalize during remission, this would support a substantial contribution from acute glycemic instability. Persistent elevation despite improved glycemia would raise the possibility of ongoing inflammatory, mitochondrial, vascular, or antioxidant-defense abnormalities.

Redox trajectories around diagnosis should be interpreted with acute metabolic status. DKA, dehydration, acidosis, infection, and acute catabolism can alter oxidative and inflammatory markers [116]. Values obtained during acute decompensation represent a stress state rather than a stable phenotype. Reassessment after metabolic stabilization provides a more reliable baseline. Serial sampling allows classification of transient redox activation, persistent redox dysregulation, or stage-dependent biomarker evolution. This approach is particularly relevant in children, where the clinical presentation at diagnosis varies widely.

Residual C-peptide provides an essential anchor for early-stage redox studies. It reflects remaining endogenous insulin secretion and β-cell functional reserve. Redox markers evaluated alongside C-peptide can clarify whether oxidative injury is associated with β-cell decline, remission duration, or metabolic instability. A profile characterized by persistent oxidative molecular injury and rapid C-peptide loss has a different biological interpretation from one characterized by preserved C-peptide and minimal oxidative damage. This distinction supports redox phenotyping as a tool for early stratification rather than a nonspecific marker of diabetes status.

### 9.3. Integration with CGM and Pediatric Glycemic Patterns

CGM has major value for pediatric redox phenotyping because it captures glycemic dynamics that HbA1c cannot resolve. HbA1c summarizes mean glycemic exposure over several weeks. It does not quantify acute excursions, time above range, time below range, nocturnal patterns, or day-to-day variability. Redox biology is responsive to dynamic metabolic shifts. CGM metrics therefore provide important temporal context for interpreting oxidative biomarkers in children and adolescents.

Time above range reflects cumulative hyperglycemic exposure. Coefficient of variation reflects glycemic instability. Time in range reflects overall glycemic stability. Postprandial excursions reflect meal-insulin matching and rapid metabolic transitions. These metrics can be linked to redox profiles in ways that HbA1c cannot [117]. A child with elevated oxidative injury markers and high glycemic variability represents a different biological state from a child with the same HbA1c but stable CGM patterns. Similarly, a child with persistent redox injury despite favorable CGM metrics suggests non-glycemic drivers such as inflammation, altered antioxidant reserve, dyslipidemia, or early tissue stress.

CGM integration also strengthens longitudinal modeling. Redox biomarkers can be related to contemporaneous glucose patterns, antecedent glycemic exposure, and subsequent metabolic outcomes. This approach would allow investigators to examine whether oxidative injury follows glycemic instability, precedes worsening control, or persists independently of glucose metrics [104]. In pediatric studies, this approach can identify redox-sensitive subgroups within clinically similar populations.

### 9.4. Pediatric Tissue Susceptibility and Early Complication Biology

Children with T1D are exposed to metabolic and inflammatory stress during periods of active growth and vascular development. This has implications for tissue susceptibility. The retina, kidney, peripheral nerves, endothelium, myocardium, and developing vasculature experience prolonged lifetime exposure beginning early in life. Redox phenotyping in pediatrics therefore has relevance beyond immediate glycemic management. It can help characterize early biological risk before clinically advanced complications emerge [121].

Renal and vascular indices are especially important in pediatric redox studies. UACR, eGFR, BP, lipid profile, arterial stiffness, endothelial function, and retinal screening data can provide tissue-level context for oxidative biomarkers. Redox markers without clinical anchors provide limited translational value. When oxidative injury markers align with early renal or vascular signals, the phenotype becomes biologically more coherent. Such profiles can support risk enrichment in longitudinal cohorts and mechanistic studies.

Neurodevelopment and autonomic function also deserve consideration in pediatric redox research. T1D during childhood occurs alongside brain maturation, sleep development, and autonomic regulation. Glycemic extremes and oxidative stress can influence neural and vascular physiology [122]. Redox biomarkers should not be overinterpreted as direct indicators of neural injury, but their relationship with hypoglycemia exposure, sleep, autonomic indices, and cognitive outcomes warrants careful investigation [123]. This expands pediatric redox phenotyping beyond classical microvascular endpoints.

### 9.5. Practical Structure of Pediatric Redox Phenotyping

A pediatric redox-phenotyping framework should integrate clinical, inflammatory, antioxidant-defense, and molecular-injury domains. The clinical domain includes age, sex, pubertal stage, disease duration, insulin regimen and dose, adiposity, blood pressure, HbA1c, continuous glucose monitoring metrics, C-peptide, lipid profile, urinary albumin-to-creatinine ratio, and estimated glomerular filtration rate. The inflammatory domain includes high-sensitivity C-reactive protein, interleukin-6, tumor necrosis factor-α, and selected cytokines. The antioxidant-defense domain includes superoxide dismutase, catalase, glutathione peroxidase, reduced glutathione, and relevant micronutrient indices. The molecular-injury domain includes malondialdehyde, F2-isoprostanes, oxidized LDL, protein carbonyls, advanced oxidation protein products, nitrotyrosine, oxidized albumin, 8-hydroxy-2′-deoxyguanosine, and 8-oxodG.

From a translational perspective, redox phenotyping may be conceptualized as a multistage analytical framework in which standardized indices of antioxidant defense and oxidative molecular injury are interpreted in conjunction with T1D stage, developmental status, metabolic context, and established clinical variables. Initial application should remain within prospective observational cohorts and biomarker-integrated clinical studies. A rigorous workflow would require sampling during metabolic stability; systematic documentation of age, pubertal stage, T1D stage and duration, residual C-peptide secretion, HbA1c, continuous glucose monitoring metrics, lipid profile, adiposity, blood pressure, renal function, inflammatory status, nutritional determinants, and recent intercurrent illness; measurement of a prespecified redox panel using analytically validated methods; and longitudinal reassessment to distinguish transient perturbations from persistent redox dysregulation.

A tiered analytical strategy may provide the most practical basis for evaluating this framework. An initial panel could combine a limited number of complementary measures representing antioxidant reserve, oxidative molecular injury, and relevant clinical modifiers. Expanded panels incorporating lipid, protein, DNA, inflammatory, mitochondrial, or tissue-associated indices could then be applied when a specific biological pathway or clinical outcome is under investigation. Interpretation should rely on internally concordant multimarker patterns, temporal reproducibility, and consistency with the clinical context, not on isolated biomarker abnormalities. Candidate profiles should subsequently undergo analytical validation, external replication, assessment of longitudinal stability, and evaluation of incremental association with clinically meaningful outcomes beyond established clinical measures. Until pediatric reference intervals, reproducible classification thresholds, and outcome-linked decision rules are established, redox phenotyping should not be used for diagnosis, prognostic classification, or treatment selection in routine clinical practice. Its present translational value lies in biological characterization, cohort enrichment, mechanistic stratification, and selection of pathway-relevant endpoints in clinical research. A transition to clinical implementation would require analytical standardization, pediatric reference intervals, reproducible longitudinal classification, demonstration of incremental clinical value, and prespecified decision rules showing how a redox result would alter monitoring or management.

### 9.6. Critical Appraisal of the Evidence Base

The evidence supporting redox phenotyping in T1D is heterogeneous with respect to study population, study design, biological matrix, analytical methodology, and clinical relevance. Pediatric human studies provide the most directly applicable evidence for this review; however, the literature remains dominated by relatively small, cross-sectional, or case–control cohorts. These studies most consistently report alterations in antioxidant enzyme activity, glutathione-dependent defense, lipid peroxidation, inflammatory mediators, and selected vascular-risk markers in children and adolescents with T1D [6,13]. Nevertheless, the direction and magnitude of these abnormalities are not uniform across studies, and differences in age, pubertal status, glycemic control, disease duration, acute metabolic status, sample compartment, preanalytical handling, and assay platform limit direct comparison. At present, pediatric studies support the presence of redox dysregulation in selected patients but do not establish validated biomarker thresholds, stable redox phenotypes, or independent prediction of β-cell decline and diabetes-related complications.

Conflicting findings are particularly evident for antioxidant-enzyme activity and glutathione-related indices. Increased SOD, catalase, or GPx activity has been interpreted as compensatory induction in some studies, whereas reduced activity in other cohorts has been attributed to enzymatic inactivation, micronutrient insufficiency, substrate depletion, glycation, or diminished antioxidant reserve. Similarly, glutathione concentrations and lipid-peroxidation markers have varied according to disease stage, glycemic control, biological matrix, analytical platform, and timing relative to diagnosis or metabolic stabilization. These inconsistencies preclude assignment of a uniform biological meaning to an isolated increase or decrease and reinforce the need for concurrent assessment of antioxidant capacity, molecular injury, clinical context, and longitudinal change.

Evidence from adult human populations provides additional support for associations between oxidative biomarkers and residual C-peptide secretion, albuminuria, endothelial dysfunction, renal outcomes, and cardiovascular risk [19,24,26,32,34]. These findings strengthen the clinical plausibility of redox-based risk assessment, although their transferability to children requires caution. Adults generally have longer disease duration, greater cumulative glycemic exposure, higher prevalence of vascular and renal abnormalities, and different developmental and cardiometabolic characteristics. Adult findings should therefore be regarded as supportive clinical evidence and not as direct validation of pediatric redox phenotypes.

Experimental studies provide the strongest mechanistic evidence linking mitochondrial reactive oxygen species generation, cytokine-mediated stress, impaired peroxide detoxification, glutathione depletion, lipid and protein oxidation, DNA injury, and NRF2-regulated responses to β-cell dysfunction and tissue injury [9,17,41,42,54,56,60]. These models clarify biological pathways and identify candidate therapeutic targets, but they do not establish the diagnostic, prognostic, or therapeutic performance of circulating biomarkers in children with T1D. Findings derived from isolated cells, human islets, or animal models should therefore be presented as mechanistic support unless corroborated by human clinical studies. The relative interpretive contribution and principal limitations of pediatric, adult, and experimental data are summarized in Table 2.

The term “redox phenotype” is used in this review only to describe an exploratory framework for organizing patterns of antioxidant defense and oxidative molecular injury. It does not denote a validated patient class. The proposed biomarker combinations have not been independently reproduced, linked to standardized classification thresholds, or shown prospectively to predict clinically meaningful outcomes. Their temporal stability, biological specificity, and external validity remain unknown. These profiles should therefore be regarded as hypothesis-generating biomarker patterns requiring formal derivation, external replication, and prospective validation before any clinical or mechanistic subgroup designation can be justified.

The clinical utility of redox biomarkers in pediatric and early-stage T1D remains unproven. Most candidate markers are influenced by developmental, metabolic, inflammatory, renal, nutritional, preanalytical, and analytical factors, including age, pubertal status, glycemic exposure, adiposity, kidney function, acute illness, micronutrient availability, specimen handling, biological matrix, and assay platform. These sources of variation limit the interpretation of isolated measurements and may contribute to the heterogeneity observed across studies. More importantly, current evidence has not established whether individual biomarkers or integrated redox panels provide independent diagnostic, prognostic, or treatment-selection value beyond established clinical measures, including HbA1c, continuous glucose monitoring metrics, residual C-peptide secretion, lipid profile, adiposity, blood pressure, urinary albumin excretion, and estimated glomerular filtration rate. Clinical validation will require prospective pediatric cohorts in which redox biomarkers are evaluated using prespecified multivariable models, compared directly with conventional risk measures, and assessed for incremental discrimination, calibration, reclassification, reproducibility, and association with clinically meaningful outcomes. Until such evidence is available, redox biomarkers should be regarded as investigational measures of biological pathway activity and not as substitutes for established clinical monitoring.

## 10. Longitudinal Prediction: Redox Trajectories, Risk Modeling, and Disease Progression

### 10.1. From Cross-Sectional Biomarkers to Dynamic Redox Trajectories

Longitudinal prediction is essential for establishing the clinical relevance of redox phenotyping in T1D. Cross-sectional biomarker differences can show that oxidative injury is present in a group, but they cannot determine whether redox abnormalities precede progression, follow metabolic instability, or identify patients at increased risk for tissue injury. A predictive framework requires repeated measurement across defined disease intervals. This approach treats redox status as a dynamic biological trajectory rather than a fixed patient attribute.

Redox trajectories can be organized around disease stage. In autoantibody-positive individuals, biomarkers can be evaluated in relation to immune activity and progression to dysglycemia [76]. During dysglycemia, redox markers can be linked to loss of metabolic tolerance. At diagnosis, measurements can characterize acute immune-metabolic stress. After stabilization, they can establish baseline redox phenotype. During partial remission, they can determine whether oxidative injury resolves with improved glycemic stability or persists despite residual β-cell function [121]. In established disease, they can be evaluated as predictors of vascular, renal, neural, and retinal risk.

Trajectory analysis can distinguish transient from persistent abnormalities, identify stage-specific patterns, and establish whether antioxidant responses precede, accompany, or follow molecular injury. Persistent abnormalities during clinically stable periods should be evaluated for greater prognostic relevance than isolated values.

### 10.2. Prediction of β-Cell Decline and Remission Duration

Residual β-cell function is a central outcome in early T1D. C-peptide decline reflects loss of endogenous insulin secretion and is associated with metabolic stability, insulin requirements, and risk of hypoglycemia. Prospective studies should determine whether redox biomarkers contribute to prediction of β-cell decline beyond established clinical variables. This requires longitudinal assessment beginning near diagnosis or during presymptomatic stages.

A redox profile associated with rapid C-peptide decline would have strong biological significance. Such a profile could include persistent oxidative molecular injury, impaired antioxidant reserve, inflammatory-redox activation, or genomic stress markers. The specific pattern matters. Inflammation-associated nitrative stress suggests immune-mediated β-cell injury. Reduced GSH with elevated molecular injury suggests insufficient antioxidant buffering. Persistent DNA oxidation suggests genotoxic stress [124]. Lipid peroxidation associated with metabolic instability suggests glucose-driven or mitochondrial redox stress. These profiles represent different biological routes to β-cell dysfunction.

Partial remission provides a clinically meaningful endpoint. Children with longer remission often show better glycemic stability and lower insulin requirements. Redox biomarkers can be examined as predictors of remission onset, duration, and decline. A persistent oxidative injury profile during early post-diagnosis care could identify patients with shorter remission or faster functional deterioration. Conversely, normalization of redox markers after stabilization could indicate reversible acute stress rather than ongoing β-cell-directed redox injury. This distinction has direct relevance for trial enrichment.

### 10.3. Prediction of Glycemic Instability and CGM Evolution

Redox biomarkers can also be studied as predictors of future glycemic instability. Oxidative stress can impair β-cell function, endothelial metabolism, inflammatory signaling, and mitochondrial efficiency. These processes can influence glucose variability, insulin sensitivity, and metabolic resilience. Longitudinal CGM data provides a detailed platform for testing these relationships.

Predictive models can relate baseline redox profiles to subsequent changes in time in range, time above range, glycemic variability, nocturnal glycemia, and hypoglycemia exposure. This approach can determine whether oxidative injury is merely associated with current glycemic instability or predicts future deterioration. A child with stable HbA1c but elevated inflammatory-redox markers could develop worsening CGM metrics over time. High glycemic variability may also arise from behavioral, dietary, treatment-related, or insulin-delivery factors independent of a persistent redox signal.

Such distinctions are important for precision care. Redox biomarkers should not be used as substitutes for CGM. They may provide complementary mechanistic context for CGM patterns. They can help separate glucose exposure, antioxidant reserve, inflammation, lipid injury, and tissue susceptibility. This approach could be evaluated for its incremental contribution to individualized risk models and for its utility as a source of mechanistic trial endpoints.

### 10.4. Prediction of Renal, Retinal, Vascular, and Neural Outcomes

The predictive value of redox phenotyping is especially relevant to complications. Traditional risk models emphasize HbA1c, disease duration, BP, lipid profile, smoking exposure, renal markers, and genetic or demographic factors. These factors are necessary but do not fully explain heterogeneity in complication susceptibility. Redox biomarkers can add information about molecular injury and tissue stress.

Renal prediction can incorporate UACR, eGFR, BP, oxidized albumin, AOPPs, 8-OHdG, inflammatory markers, and antioxidant-defense indices. Persistent protein or DNA oxidation in association with early renal signals could identify a candidate renal redox-risk pattern. Retinal prediction can incorporate glycemic exposure, BP, lipid profile, inflammatory markers, endothelial markers, and oxidative injury markers [125]. Vascular prediction can integrate oxidized LDL, nitrotyrosine, AOPPs, arterial stiffness, endothelial function, and lipid parameters. Neural prediction can explore relationships among redox markers, glycemic extremes, autonomic function, neuropathic symptoms, and neurocognitive measures [126].

These applications require repeated biomarker measurement and clinically meaningful endpoints. The objective is not to assign causality from a single marker. The objective is to develop redox-informed risk models that improve prediction beyond established variables. A patient with modest HbA1c elevation but persistent inflammatory protein oxidation and vascular redox abnormalities could represent a potentially higher-risk biological profile requiring prospective validation. Longitudinal studies are needed to quantify this added predictive value.

### 10.5. Biomarker Modeling and Phenotype Stability

Redox phenotyping requires statistical approaches that can handle correlated biomarkers, repeated measurements, and heterogeneous trajectories. Single-marker thresholds are unlikely to capture biology adequately. Composite scores, latent class models, trajectory clustering, principal component approaches, and machine-learning methods can identify reproducible redox patterns. These methods should be anchored to biological interpretation. A mathematically derived cluster must correspond to plausible oxidative defense, molecular injury, inflammatory activation, or tissue-risk biology.

Phenotype stability is a key question. Some redox phenotypes will be transient and context-dependent. Others will persist across time and indicate stable susceptibility. A transient phenotype can occur during DKA, infection, rapid pubertal growth, or short periods of poor glycemic control. Longitudinal studies should determine whether persistent candidate redox patterns reflect chronic inflammatory-redox activation, reduced antioxidant reserve, recurrent glycemic variability, dyslipidemia, or early tissue injury.

Time alignment is essential. Redox biomarkers should be linked to antecedent glycemic exposure, contemporaneous clinical state, and subsequent outcomes. For example, CGM data from a prespecified antecedent interval can provide context for oxidative markers measured at a clinic visit. C-peptide trajectories can be linked to redox profiles over months. Complication markers can be evaluated over years. This temporal structure strengthens inference and reduces misclassification.

## 11. Therapeutic Implications: Phenotype-Directed Antioxidant, Mitochondrial, Anti-Inflammatory, and NRF2-Focused Strategies

The therapeutic implications of redox phenotyping are currently conceptual and investigational. Biomarker-guided antioxidant, mitochondrial-targeted, anti-inflammatory, and NRF2-directed interventions are scientifically plausible, but available evidence does not support their routine clinical use in T1D, particularly in children. Most candidate approaches have been evaluated in experimental models, mechanistic studies, or limited clinical cohorts, and their efficacy, safety, optimal timing, and long-term effects in pediatric T1D remain uncertain. Redox biomarkers may support participant enrichment, mechanistic stratification, and target-engagement assessment in clinical trials; however, they should not currently be used to direct treatment outside research settings. The intervention and corresponding endpoints should be aligned with the putative biological pathway, and lipid-peroxidation, protein-oxidation, genomic-injury, antioxidant-defense, mitochondrial, and inflammation-associated profiles should be evaluated through rigorously designed biomarker-enriched studies.

### 11.1. Pathway-Aligned Investigational Strategies

The principal translational value of redox profiling is the alignment of an investigational intervention with evidence of involvement of the corresponding biological pathway. Candidate approaches include the optimization of glycemic stability and nutritional status, restoration of glutathione-dependent or enzymatic antioxidant capacity, reduction in lipid and vascular oxidative injury, modulation of inflammation-associated redox activity, and investigation of mitochondrial- or NRF2-directed strategies. These approaches should not be inferred from T1D diagnosis alone. Their evaluation requires prespecified evidence of pathway activation, analytically appropriate biomarkers, clinically relevant safety monitoring, and endpoints that reflect both target engagement and disease-related outcomes.

Interpretation should remain conservative. Reduced glutathione availability or impaired peroxide detoxification may justify investigation of antioxidant-defense pathways, whereas persistent lipid peroxidation may support evaluation of metabolic or vascular mechanisms. Coexisting cytokine activation, nitrative injury, or protein oxidation may identify inflammation-associated redox activity, while mitochondrial or NRF2-related interventions require direct pathway-level evidence. None of these biomarker patterns currently define treatment indication. Their present role is limited to mechanistic stratification and participant enrichment within rigorously designed clinical studies, particularly in pediatric populations.

### 11.2. Biomarker-Enriched Trial Design

Redox phenotyping has its strongest therapeutic value in trial design. Biomarker-enriched trials can select participants with measurable pathway activation, match interventions to redox phenotype, and use mechanistic endpoints that correspond to the intervention. This increases interpretability and reduces dilution from participants without the targeted biology. It also allows stratified analysis by disease stage, age, pubertal status, C-peptide, CGM pattern, lipid profile, and inflammatory state.

Trial design should align phenotypes, mechanisms, and endpoints. A GSH-deficient phenotype requires GSH- and GPx-related endpoints. A lipid peroxidation phenotype requires MDA, F2-isoprostanes, oxidized LDL, and vascular markers. A protein oxidation phenotype requires protein carbonyls, AOPPs, nitrotyrosine, oxidized albumin, and renal or endothelial endpoints. A DNA oxidation phenotype requires 8-OHdG or 8-oxodG with renal and repair context. An immune-redox phenotype requires cytokine, nitrative, and oxidative injury endpoints. This structure makes biological interpretation possible even when clinical outcomes require longer follow-up.

Pediatric trials should prioritize safety, feasibility, and developmental appropriateness. Blood volume, visit burden, pubertal stage, adherence, family context, and psychosocial factors affect study design. Non-invasive urinary biomarkers, CGM-derived endpoints, and clinically obtained blood samples can reduce burden. Redox biomarkers should be integrated into existing pediatric workflows when possible. This improves translational feasibility and supports future clinical implementation.

## 12. Conclusions and Future Directions

Oxidative stress may constitute a quantifiable component of biological heterogeneity in T1D; however, clinically validated redox phenotypes have not yet been established. The framework advanced in this review is explicitly exploratory and integrates indices of antioxidant defense, oxidative molecular injury, inflammation-associated redox activity, and tissue-relevant dysfunction to define candidate patterns of redox dysregulation. These patterns should be regarded as provisional analytical constructs, not as established phenotypes, since reproducible classification thresholds, temporal stability, external replication, and prospective associations with clinically meaningful outcomes remain undetermined. Their utility for patient classification, prognostic assessment, and therapeutic stratification therefore requires rigorous evaluation in longitudinal, independently validated cohorts.

A prerequisite for this framework is rigorous and consistent definition of the study population. Presymptomatic stage 1 and stage 2 T1D, newly diagnosed stage 3 T1D, the partial-remission phase, and short-duration established T1D constitute clinically and biologically distinct disease states and should not be combined without prespecified stage-specific or stage-stratified analyses. Future studies should systematically report islet autoantibody status, glycemic stage, interval from clinical diagnosis, metabolic stability at the time of sampling, partial-remission status, residual C-peptide secretion, and total disease duration. Such characterization is essential for assigning redox biomarker findings to the appropriate phase of T1D and for enabling valid comparisons across cohorts.

Future studies should evaluate whether reproducible, integrated redox patterns can be derived and validated, instead of examining isolated biomarkers alone. SOD, CAT, GPx, GSH, MDA, F2-isoprostanes, oxidized LDL, protein carbonyls, AOPPs, nitrotyrosine, oxidized albumin, 8-OHdG, 8-oxodG, hsCRP, IL-6, and TNF-α should be interpreted with HbA1c, CGM metrics, C-peptide, lipid profile, BP, UACR, eGFR, developmental stage, and disease duration. This structure can distinguish antioxidant-defense impairment, lipid-centered oxidation, inflammatory protein injury, genomic oxidative burden, vascular-redox activation, and renal redox stress. No single marker is currently sufficient to define clinically meaningful redox status. Classification requires biological integration and clinical anchoring.

Methodological standardization is essential for reproducibility. Studies should harmonize sample matrix, processing time, storage conditions, hemolysis criteria, fasting status, acute illness status, assay platform, normalization strategy, and reporting units. Enzyme activity, protein abundance, transcript abundance, and metabolite concentration should be reported as distinct analytical outputs. MDA assays should be distinguished from TBARS-based methods. F2-isoprostanes, oxidized LDL, AOPPs, nitrotyrosine, 8-OHdG, and 8-oxodG require explicit reporting of matrix, platform, calibration, and quality control. Pediatric reference intervals should account for age, sex, pubertal stage, renal function, and clinical stability.

Longitudinal pediatric cohorts are required to define temporal and prognostic significance. Serial sampling should extend from autoantibody positivity, dysglycemia, diagnosis, or early post-diagnosis through stabilization, partial remission, puberty, and early complication surveillance. This design can separate acute redox activation during DKA, infection, or metabolic decompensation from persistent redox dysregulation during stable disease. C-peptide trajectories can clarify relationships with β-cell decline and remission duration. CGM metrics can define links with time in range, time above range, glycemic variability, and postprandial excursions. Renal, retinal, vascular, and neural outcomes should be used to determine whether candidate redox patterns provide incremental predictive information beyond conventional clinical variables.

Mechanistic validation should accompany cohort development. Peripheral blood and urine markers provide accessible measures, but they do not establish tissue origin. Cellular assays, leukocyte redox profiling, endothelial functional studies, metabolomics, proteomics, lipidomics, transcriptomics, mtDNA-focused analyses, and DNA repair assessments can strengthen biological interpretation. Islet-specific conclusions should be supported by experimental data, human islet studies, or longitudinal associations with C-peptide dynamics. NRF2-related studies should include pathway-level readouts involving GSH synthesis, detoxification enzymes, peroxide-handling systems, and oxidative injury markers.

Therapeutic translation should proceed through biomarker-enriched trials. Future biomarker-enriched trials may evaluate participant selection according to prespecified candidate redox profiles, once those profiles have undergone analytical and clinical validation. Phenotype and endpoint alignment are necessary for mechanistic interpretation. GSH-related profiles require GSH and GPx endpoints. Lipid oxidation profiles require MDA, F2-isoprostanes, oxidized LDL, and vascular measures. Protein oxidation profiles require protein carbonyls, AOPPs, nitrotyrosine, oxidized albumin, and renal or endothelial indices. Genomic injury profiles require 8-OHdG or 8-oxodG with renal and repair context. Inflammatory-redox profiles require hsCRP, IL-6, TNF-α, nitrative stress markers, and oxidative molecular injury endpoints. Antioxidant, mitochondrial, anti-inflammatory, and NRF2-focused strategies should be evaluated only when the target pathway is demonstrably involved.

Clinical implementation will require validated decision thresholds from longitudinal outcome-linked cohorts. A future redox score would ideally distinguish persistent oxidative molecular injury, antioxidant-defense impairment, inflammatory-redox activation, vascular-redox patterns, renal redox stress, and absence of measurable redox injury. The absence of measurable pathway activation may also prove informative by identifying individuals less likely to benefit from pathway-directed antioxidant approaches. This hypothesis requires prospective testing. A tiered strategy is the most practical path forward. Minimal panels can support large cohorts. Expanded panels can support mechanistic studies and trials. Multi-omics and cellular assays can be reserved for specialized research settings.

The central objective is to determine which patients have measurable oxidative injury, which pathway predominates, whether the phenotype predicts progression or complications, and whether pathway-directed intervention modifies the phenotype. Achieving this objective requires standardized assays, longitudinal pediatric cohorts, multimodal risk modeling, mechanistic validation, and phenotype-enriched intervention studies. These advances will be required to determine whether redox profiling can progress from a hypothesis-generating research framework to a reproducible approach for biological stratification, outcome prediction, or intervention studies in T1D.

## Figures and Tables

**Table 1:** Redox biomarker domains in pediatric and early-stage type 1 diabetes.

Domain	Biomarkers	Biological Axis	Provisional Biological Interpretation	Methodological Caveats	References
Antioxidant enzymes	Superoxide dismutase, catalase, glutathione peroxidase, glutathione reductase, peroxiredoxins, thioredoxin	Superoxide and peroxide detoxification	Compensatory antioxidant induction, enzymatic insufficiency, or peroxide-handling failure	• Enzyme activity and abundance are not interchangeable • Values are affected by hemolysis, sample matrix, glycation, micronutrient status, storage, and assay platform • Interpretation requires concurrent oxidative damage, inflammatory, metabolic, and renal context	[10,27,63]
Glutathione system	Reduced glutathione, oxidized glutathione, reduced-to-oxidized glutathione ratio, glutathione peroxidase, glutathione reductase	Thiol buffering, peroxide detoxification, and hydroperoxide reduction	Redox reserve, substrate depletion, impaired antioxidant recycling, or glutathione-dependent vulnerability	• Requires rapid processing and strict preanalytical control • Interpretation depends on selenium status, NADPH availability, inflammation, and metabolic state • Glutathione peroxidase activity should be interpreted with glutathione status and relevant micronutrients	[27,64]
Lipid oxidation	Malondialdehyde, F2-isoprostanes, oxidized low-density lipoprotein, lipid hydroperoxides, 4-hydroxynonenal	Membrane, lipid-aldehyde, and lipoprotein peroxidation	Lipid-peroxidation phenotype or vascular–lipoprotein redox injury	• Malondialdehyde assay specificity is variable • F2-isoprostanes require rigorous analytical methods • Oxidized low-density lipoprotein assays are epitope-dependent • Lipid oxidation markers require interpretation with lipid profile, glycemic variability, inflammation, and vascular context	[65,66,67]
Protein oxidation	Protein carbonyls, advanced oxidation protein products, oxidized albumin, thiol loss	Structural protein modification and extracellular redox buffering	Sustained macromolecular oxidation, carbonyl stress, inflammatory protein injury, or impaired extracellular antioxidant capacity	• Influenced by albumin concentration, renal handling, inflammation, acute illness, and assay chemistry • Persistent abnormalities are more informative than isolated values during infection or metabolic decompensation • Interpretation is strengthened by renal, endothelial, and inflammatory markers	[68,69,70]
Nitrative stress	Nitrotyrosine, peroxynitrite-related adducts, nitric oxide-derived protein modifications	Nitric oxide–superoxide interaction and reactive nitrogen species-mediated injury	Endothelial, inflammatory, mitochondrial, or cytokine-linked nitrative phenotype	• Requires interpretation with cytokines, endothelial markers, and oxidative protein markers • Nitrotyrosine should not be treated as a nonspecific oxidative stress marker • Best used in panels that include inflammatory and vascular-redox indices	[44,71,72]
DNA oxidation	8-hydroxy-2′-deoxyguanosine, 8-oxodG, leukocyte DNA oxidation, mitochondrial DNA damage indices	Nuclear and mitochondrial genomic injury with repair-associated oxidative turnover	Genotoxic redox burden, mitochondrial oxidative injury, or repair-linked oxidative stress	• Matrix-dependent and not tissue-specific • Urinary values require renal and hydration context • Interpretation should include renal function, inflammation, antioxidant status, and clinical stability • Compartment-specific assays are required to distinguish nuclear from mitochondrial DNA injury	[73,74]
Inflammation–redox coupling	High-sensitivity C-reactive protein, interleukin-6, tumor necrosis factor alpha, interleukin-1 beta, interferon gamma, myeloperoxidase, adhesion molecules	Immune-redox amplification and cytokine-associated oxidative injury	Inflammation-dominant redox phenotype, immune-metabolic activation, or cytokine-linked oxidative and nitrative injury	• Confounded by infection, puberty, adiposity, autoimmunity, and acute metabolic instability • Persistent co-elevation with oxidative injury markers is more informative than isolated cytokine elevation • Should be interpreted with glycemic, developmental, vascular, and renal variables	[75,76,77]
Clinical modifiers	Hemoglobin A1c, continuous glucose monitoring variability, time in range, C-peptide, lipid profile, body mass index, blood pressure, urinary albumin-to-creatinine ratio, estimated glomerular filtration rate	Metabolic, vascular, renal, developmental, and beta-cell context	Distinguishes transient redox response from persistent dysregulation and supports longitudinal risk stratification	• Required for biological interpretation • Isolated redox biomarkers should not be overinterpreted • Longitudinal sampling is preferable to single time-point classification • Pediatric interpretation should account for age, sex, puberty, renal function, and clinical stability	[16,78,79]

Abbreviations: 8-oxodG, 8-oxo-7,8-dihydroguanosine; NADPH, nicotinamide adenine dinucleotide phosphate.

**Table 2:** Considerations for interpreting redox biomarkers in type 1 diabetes.

Data Source	Contribution	Limitations	Interpretation	References
Pediatric prospective or longitudinal studies	Temporal relationships with glycemic variability, C-peptide, vascular function, or disease progression	Limited cohort size and repeated biomarker sampling	Highest relevance for pediatric prognostic evaluation	[6,13,26]
Pediatric cross-sectional or case–control studies	Identification of biomarker differences and candidate associations	No assessment of temporality or phenotype stability	Association-generating evidence	[11,18,27]
Adult T1D cohorts	Long-term relationships with renal, vascular, and microvascular outcomes	Different disease duration and cardiometabolic context	Supportive data requiring cautious pediatric extrapolation	[19,21,22,25]
Mixed-age clinical studies	Broader clinical context and biomarker–outcome associations	Pediatric effects may be obscured in pooled analyses	Pediatric evidence only when age-stratified findings are reported	[10,24]
Human islet or primary-cell studies	β-cell-specific or immune-cell mechanistic evidence	Limited representation of systemic disease biology	Mechanistic plausibility only	[9,17,30,31,39]
Animal and in vitro models	Pathway identification and intervention testing	Species, dose, tissue, and model dependence	Hypothesis generation, not clinical validation	[14,33,41]

## Data Availability

No new data were created or analyzed in this study. Data sharing is not applicable to this article.
